# Serum Mg Isotopic Composition Reveals That Mg Dyshomeostasis Remains in Type 1 Diabetes despite the Resolution of Hypomagnesemia

**DOI:** 10.3390/ijms242115683

**Published:** 2023-10-27

**Authors:** Kaj Vaughan Sullivan, Yasmina Assantuh, Rosa Grigoryan, Marta Costas-Rodríguez, Eduardo Bolea-Fernandez, Bruno Lapauw, Steven Van Laecke, Frank Vanhaecke

**Affiliations:** 1 Atomic and Mass Spectrometry—A&MS Research Unit, Department of Chemistry, Ghent University, 9000 Ghent, Belgium; kaj.sullivan@ugent.be (K.V.S.);; 2 The Isotoparium, Division of Geological and Planetary Sciences, California Institute of Technology, Pasadena, CA 91125, USA; 3 Centro de Investigación Mariña, Departamento de Química Analítica y Alimentaria, Grupo QA2, Universidade de Vigo, 36310 Vigo, Spain; 4 Department of Analytical Chemistry, Aragón Institute of Engineering Research (I3A), University of Zaragoza, 50009 Zaragoza, Spain; 5 Department of Endocrinology, Ghent University Hospital, 9000 Ghent, Belgium; 6 Department of Internal Medicine and Pediatrics, Ghent University, 9000 Ghent, Belgium; 7 Renal Division, Department of Internal Medicine and Pediatrics, Ghent University Hospital, 9000 Ghent, Belgium

**Keywords:** magnesium, type 1 diabetes mellitus, MC-ICP-MS, hypomagnesemia, stable isotope ratios

## Abstract

Hypomagnesemia was historically prevalent in individuals with type 1 diabetes mellitus (T1DM), but contemporary results indicate an incidence comparable to that in the general population, likely due to improved treatment in recent decades, resulting in better glycemic control. However, a recent study found a significant difference between the serum Mg isotopic composition of T1DM individuals and controls, indicating that disruptions to Mg homeostasis persist. Significant deviations were also found in samples taken one year apart. To investigate whether the temporal variability in serum Mg isotopic composition is linked to the transient impact of administered insulin, Mg isotope ratios were determined in serum from 15 T1DM individuals before and one hour after insulin injection/meal consumption using multi-collector inductively coupled plasma-mass spectrometry. Consistent with results of the previous study, significant difference in the serum Mg isotopic composition was found between T1DM individuals and 10 sex-matched controls. However, the average difference between pre- and post-insulin injection/meal T1DM samples of 0.05 ± 0.13‰ (1SD) was not significant. No difference was observed for controls before (−0.12 ± 0.16‰) and after the meal (−0.10 ± 0.13‰) either, suggesting a lack of a postprandial Mg isotopic response within one hour of food consumption, and that the timing of the most recent meal may not require controlling for when determining serum Mg isotopic composition.

## 1. Introduction

Diabetes mellitus is a metabolic disorder that is typically characterized by the loss of the ability of β-pancreatic cells to adequately produce and secrete insulin, leading to hyperglycemia [1]. According to a World Health Organization (WHO) report from 2018, diabetes mellitus is responsible for an estimated 1.5 million deaths annually, making it the eighth most fatal condition in the world [2]. In type 2 diabetes mellitus (T2DM), which is linked to genetics and lifestyle habits, cells do not respond adequately to insulin (insulin resistance), whereas type 1 diabetes mellitus (T1DM) is caused by an autoimmune response which attacks insulin-producing cells (β-cells) [1,3]. This triggers an absolute insulin deficiency, which necessitates the administration of exogenous insulin to maintain glucose homeostasis [4,5]. A common risk presented in individuals with T1DM is hypoglycemia, which can be consequence of strict glycemic control through treatment with insulin. Low blood glucose levels can lead to complications and can also be life-threatening—especially in the case of nocturnal hypoglycemia [4,5].

In the 1980s and early 1990s, hypomagnesemia (serum Mg levels < 17 mg L^−1^) was commonly reported in individuals with T1DM [6,7,8]. For example, in 1982, McNair et al. reported a high prevalence of hypomagnesemia (38%) and the inverse correlation of serum Mg concentration and glucose level [7]. This may be due to insulin playing a critical role in glucose and lipid metabolism and potentially modulating Mg transport from the extracellular to the intracellular space [9,10]. However, more recent studies have reported the prevalence of hypomagnesemia to be as low as 2.9% in cohorts of individuals with T1DM, which is comparable to the general population [8,11,12]. This has been attributed to the significantly improved treatment of T1DM in recent decades, resulting in better glycemic control [8]. Indeed, in a longitudinal study of 27,035 Austrians and Germans with T1DM, the proportion with good glycemic control (HbA1c ≤ 7.5%) rose from 25 to 45% between 1995 and 2004, while the proportion with poor glycemic control decreased from 40 to 16% [13]. However, the link between hypomagnesemia and poor glycemic control in individuals with T1DM is still being explored.

A 2020 systematic review and meta-analysis found an association between reduced serum Mg levels and poor glycemic control in T1DM, but results were conflicting [14]. A recent investigation found that this association may be limited to insulin-resistant individuals only, whereas another found that there may even be no association at all, going so far as to suggest that hypomagnesemia is currently not a relevant topic in routine care for individuals with T1DM [8,11,14]. However, Mg status is difficult to assess, possibly due to blood accounting for just ~1% of bodily Mg, with the rest being stored in muscle (27%), bone (53%), and other tissues (19%) [15,16,17,18]. Erythrocyte Mg concentrations have also been shown to be significantly lower in individuals with T1DM than controls, even in the absence of a difference between their plasma Mg concentrations, and this was attributed to urinary Mg loss [19]. In blood serum, about 55% of Mg exists as a free ion (iMg^2+^), this being the physiologically-active fraction, whereas 32% is protein-bound (predominantly to albumin), and 13% is in anionic complexes [20,21]. The Mg in blood and tissue are in a constant state of exchange, with the kidneys being capable of filtering up to 2400 mg of Mg per day [22]. The much larger exchangeable pool of Mg in muscle and other tissues is often drawn from to maintain a tight range of serum Mg concentrations, which can easily mask deficiency [15,23,24]. There may also be other potential markers of Mg status to consider. For example, the study of Grigoryan et al., which reported no difference between the serum Mg concentration of individuals with T1DM and healthy controls, found a significant difference between their serum Mg isotopic compositions [25]. Taken together, this evidence indicates that the disruption of Mg homeostasis remains a relevant topic in T1DM despite the reduced incidence of hypomagnesemia (as defined by serum Mg concentrations).

Magnesium has three naturally-occurring stable isotopes: ^24^Mg ^25^Mg, and ^26^Mg, with relative natural abundances of 78.99, 10.00, and 11.01%, respectively [26]. The tight regulation of blood serum Mg concentration (among other essential mineral elements) highlights a significant limitation of markers based on metal abundances alone. However, stable isotope ratios of essential mineral elements (e.g., ^26^Mg/^24^Mg, ^65^Cu/^63^Cu, and ^66^Zn/^64^Zn) can often reflect changes in metal homeostasis associated with a particular pathological condition when metal abundances do not [25,27,28]. For example, the stable Mg isotopic compositions (but not Mg concentration) of plasma, liver, kidney, and heart of obese minipigs are significantly different (relatively enriched in ^24^Mg) from those in lean minipigs [28]. Magnesium isotope ratio data also indicated that Mg metabolism is already disrupted in the earliest stages of deteriorating glucose metabolism, and it was suggested that the results obtained by Grigoryan et al. may not be specific to the T1DM condition, but instead to phenotypes associated with insulin resistance preceding clinical disease conditions [25,28]. However, in Grigoryan et al., a substantial difference (~1‰—the magnitude encompassing almost the entire range of δ^26^Mg values determined across the organs and tissues of a mammal) was also observed in the serum Mg isotopic compositions of the same T1DM individuals one year apart [25,29,30]. In the present study, the effect of administered insulin was investigated as a cause of temporal variability in the serum Mg isotopic composition through the comparison of blood serum samples collected before and one hour after insulin injection and meal consumption by T1DM individuals. The serum Mg isotopic response of healthy individuals after meal consumption was also examined.

## 2. Results

### 2.1. Quality Assurance/Quality Control

The quality of the sample digestion and the accuracy of Mg concentration measurements were monitored using the Seronorm™ Trace Elements Serum L-1 reference material (lot 1801802, SERO AS, Billingstad, Norway). As can be seen in Table 1, the Mg concentration was within the acceptable range provided for this reference material (18.6 to 21.6 mg L^−1^). The reproducibility and quality of the chromatographic procedure were monitored by processing the serum reference material alongside samples. Instrument performance and the accuracy of Mg isotope ratio measurements were monitored by the measurement of the Mg isotopic composition of a CPI Mg standard (CPI International), which agreed with previously reported data (Table 1). Magnesium isotopic analysis of the serum reference material yielded values that agreed with previously reported results (Table 1). The contribution of the Mg procedural blank was below 4% of the Mg signal intensity of samples.

Isotopic data are reported in delta (δ) notation relative to the international reference material, DSM3, where R is the ratio of the signal of ^*x*^*Mg* (^25^Mg or ^26^Mg) to ^24^Mg (Equation (1)).

(1) $$\delta^{x/24}{Mg}_{sample}=\left(\frac{R_{sample}^{x/24}}{R_{DSM3}^{x/24}}-1\right)\cdot 1000$$

**Figure 1:**
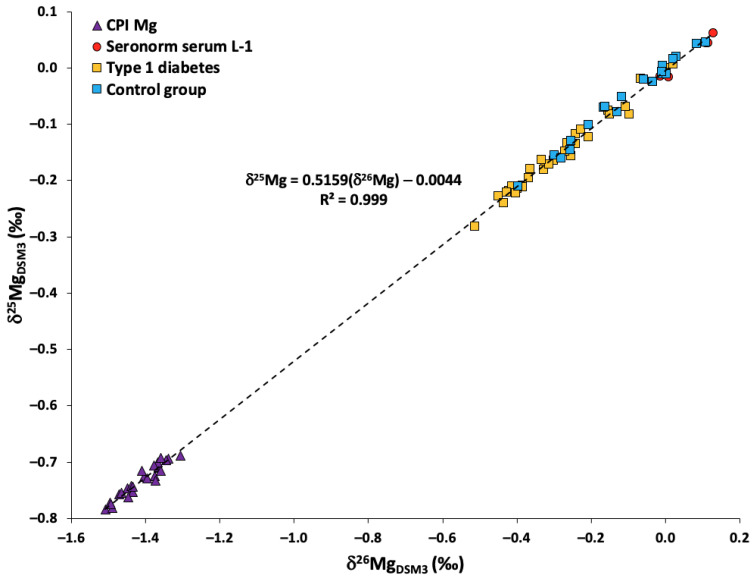
Bivariate (3-isotope) Mg isotope ratio plot for Mg isotope ratio data obtained for CPI Mg, Seronorm serum L-1, and blood serum samples from control group and individuals with T1DM.

The slope of the fractionation line produced by the δ^26^Mg and δ^25^Mg values of all samples, standards, and reference materials analyzed for this study was within the range defined by kinetically (slope = 0.5110) and thermodynamically (slope = 0.5210) governed mass-dependent fractionation (Figure 1) [32]. This indicates the absence of resolvable polyatomic interferences or mass-independent isotope effects at the level of the precision attained.

### 2.2. Serum Mg Concentration and Clinical Parameters

**Figure 2:**
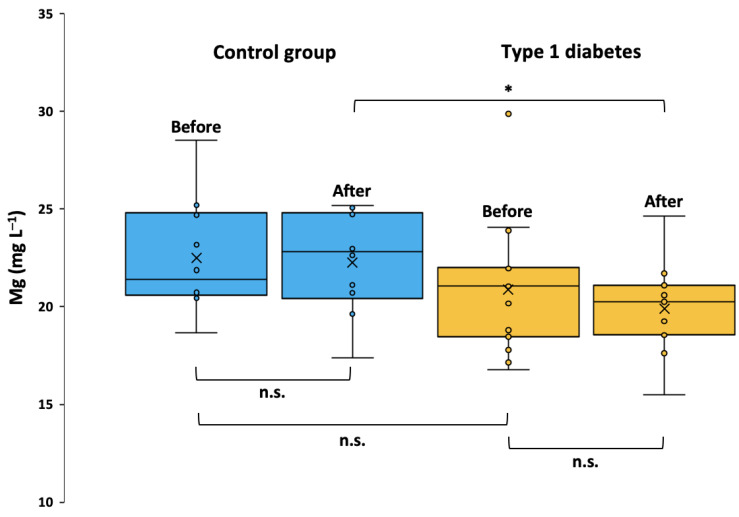
Magnesium concentrations determined in blood serum samples drawn from control group and T1DM individuals before and one hour after insulin injection and/or consuming the standard meal. The box represents the 25–75th percentiles (with the median as a horizontal line, mean as a cross) and the whiskers represent the range. Outliers are denoted outside of the range if they exceed a distance of 1.5 times the interquartile range below the first quartile or above the third quartile. The threshold for significance was defined as *p* < 0.05 (*), n.s. means not significant. All other relationships displayed no statistical significance (*p* ≥ 0.05).

The serum Mg concentration was determined in 20 serum samples (10 taken before meal, 10 taken after) from 10 healthy controls, and in 30 serum samples (15 taken before insulin injection/meal consumption, 15 taken after) from 15 individuals with T1DM (Table 2, Figure 2). No difference (*p* = 0.79) was observed between the serum Mg concentration before (mean = 22.5 ± 2.9 mg L^−1^) and after the meal (mean = 22.2 ± 2.5 mg L^−1^) in healthy controls, suggesting a lack of a postprandial response within one hour of food consumption. Also, no difference (*p* = 0.23) was observed in the diabetic group between samples taken before (mean = 20.9 ± 3.4 mg L^−1^) and one hour after insulin administration and consumption of the meal (mean = 19.9 ± 2.2 mg L^−1^). Seven out of 14 T1DM individuals in which HbA1c was determined had good glycemic control (HbA1c ≤ 7.5%), which is in-line with recent trends [13]. However, there was no correlation between glycemic control and serum Mg concentration (*r* = 0.33, *p* = 0.25). Data were not collected for insulin resistance markers (triglycerides/high-density lipoprotein (TG/HDL) ratio, TG levels, and total cholesterol/HDL ratio), but individuals with T1DM required only 0.04 to 0.42 insulin units (IU; 1 IU = 34.7 μg of pure crystalline insulin) per kg of body weight ahead of meal consumption, with a median of 0.10 IU kg^−1^. The daily insulin dosage for participants of this study was not available, but a daily dose of >1.5 IU kg^−1^ is considered to be suggestive of insulin resistance [33,34].

The serum Mg concentration is significantly lower in the diabetic group than in the control group when comparing the post-meal values (*p* = 0.021). The serum Mg concentration is also lower in the pre-meal samples from individuals with T1DM, but this difference failed to reach significance (*p* = 0.23). Despite the generally lower serum Mg concentration in T1DM individuals as compared to healthy controls, only one out of 15 (6.7%) T1DM individuals in this study had a serum Mg concentration that was consistently in the range of hypomagnesemia (<17.0 mg L^−1^) [35,36]. No control group participants had a value below 17.0 mg L^−1^. These results agree with other recent studies reporting a similar prevalence of hypomagnesemia in T1DM as compared to the general population [8,11,12].

### 2.3. Serum Mg Isotopic Composition

**Figure 3:**
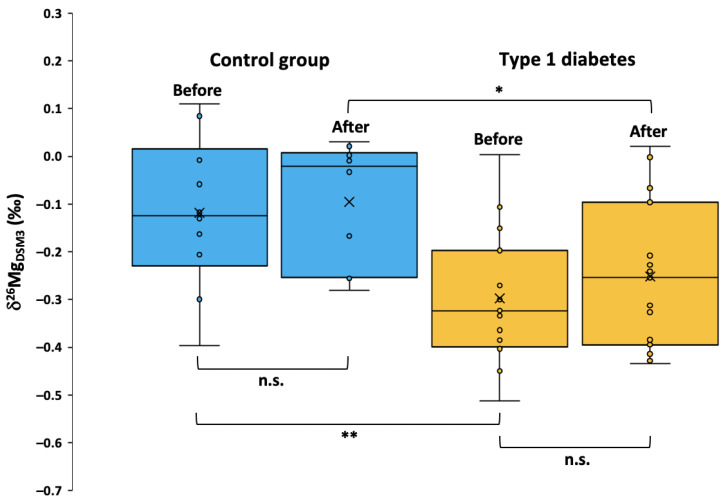
δ^26^Mg values determined in blood serum samples drawn from control group and T1DM individuals before and one hour after insulin injection and/or consuming the standard meal. The box represents the 25–75th percentiles (with the median as a horizontal line, mean as a cross) and the whiskers represent the range. Outliers are denoted outside of the range if they exceed a distance of 1.5 times the interquartile range below the first quartile or above the third quartile. The thresholds for significance were defined as *p* < 0.05 (*) and *p* < 0.01 (**), n.s. means not significant. All other relationships displayed no statistical significance (*p* ≥ 0.05).

The serum Mg isotopic composition was determined in samples provided by healthy controls before and one hour after meal consumption, and in samples from T1DM individuals before and one hour after insulin injection/meal consumption (Table 3, Figure 3). For healthy controls, no difference (*p* = 0.49) was observed between the serum Mg isotopic composition before (mean δ^26^Mg = −0.12 ± 0.16‰) and after the meal (mean δ^26^Mg = −0.10 ± 0.13‰), suggesting a lack of a postprandial Mg isotopic response within one hour of food consumption. This indicates that as is the case for Cu and Zn, the timing of the most recent meal may not require controlling for when determining serum Mg isotopic composition [37,38,39]. Although two T1DM individuals had δ^26^Mg values that were 0.27 and 0.30‰ higher following insulin injection/meal consumption, no difference (*p* = 0.18) was observed overall in the T1DM group between samples taken before (mean δ^26^Mg = −0.30 ± 0.14‰) and after the meal (mean δ^26^Mg = −0.25 ± 0.15‰). However, δ^26^Mg values were significantly lower in the T1DM group than in the control group when comparing both the respective pre-insulin injection and/or meal (*p* = 0.0068) and post-insulin injection and/or meal values (*p* = 0.027). This is in agreement with previous results and provides additional evidence of the disruption of Mg homeostasis in T1DM, even with rates of hypomagnesemia (as defined by serum Mg concentration) that are largely similar to those of the general population [25].

## 3. Discussion

### 3.1. Potential Mechanisms behind Large Mg Isotopic Variations in T1DM

Blood serum samples collected before and one hour after insulin injection and meal consumption by T1DM individuals were analyzed to investigate whether previously identified temporal variability in the serum Mg isotopic composition (~1‰ in serum samples from the same T1DM individuals one year apart) is related to the temporary effect of administered insulin [25]. In Grigoryan et al., the serum δ^26^Mg values for T1DM ranged from −1.80 to −0.22‰ (on average 0.65‰ lower than that of the reference population), and in the one-year follow-up, the serum δ^26^Mg values for T1DM ranged from −1.54 to −0.32‰ (on average 0.75‰ lower). However, for some T1DM individuals, large differences were observed between the serum δ^26^Mg values for the two sampling events (up to 1.04‰). Whereas the relative enrichment of ^24^Mg in T1DM compared to controls observed in Grigoryan et al. was replicated, a significantly reduced magnitude of difference was observed in the present study. This is despite the pre- and post-meal serum δ^26^Mg values for controls being statistically indistinguishable (*p* = 0.92 and *p* = 0.41, respectively) from the control group of Grigoryan et al. [25]. In the present study, the average serum δ^26^Mg value for pre-injection/meal T1DM samples was 0.18‰ lower than in healthy controls (*p* = 0.0068), and for the post-injection/meal samples, the T1DM average was 0.15‰ lower (*p* = 0.027). The second blood sample was drawn one hour after finishing the meal in order to integrate both the rise in glycemia due to gastro-intestinal absorption and the effects of insulin, but this had little effect on the serum Mg concentration or isotopic composition. The greatest difference observed between serum δ^26^Mg values from any sample pair from T1DM individuals was 0.30‰, but the average difference between post-insulin injection/meal and pre-insulin injection/meal samples was 0.05 ± 0.13‰ (1SD), which is not significant (*p* = 0.18).

As opposed to the 50% of T1DM individuals from this study that were determined to have good glycemic control (HbA1c ≤ 7.5%), just three out of 15 (~20%) T1DM individuals from Grigoryan et al. in whom HbA1c was determined had good glycemic control. However, in Grigoryan et al., HbA1c and Mg isotopic compositions did not show a significant correlation, even when considering only individuals with poor glycemic control. Further, HbA1c varied little between samples collected one year apart, yet the serum δ^26^Mg values varied greatly in some cases. For example, a participant with good glycemic control consistently displaying 6.2% for HbA1c in year one and year two had serum δ^26^Mg values of −1.31 and −0.27‰, respectively. Conversely, a participant with poor glycemic control, measuring 10.6% for HbA1c in year two (no measurement available for year one) had serum δ^26^Mg values of −0.47‰ in year one and −1.51‰ in year two. Taken together, this information suggests that poor glycemic control is not a cause of large serum Mg isotopic variations in T1DM.

Insulin and glucose have been demonstrated to have opposing effects on intracellular Mg^2+^, and may represent two limbs of a biphasic intracellular Mg^2+^ regulatory system, with insulin increasing the concentration of intracellular Mg^2+^ [10]. With the consistent sampling conditions of the present study failing to capture the large serum Mg isotopic variations and the timing of the previous insulin dose and meal ahead of sampling for the study of Grigoryan et al. unknown, it remains possible to speculate that sampling during the period between insulin injection (when intracellular Mg^2+^ is increased—possibly due to the inhibitory effect of insulin on the mechanism responsible for Mg^2+^ efflux) and meal consumption may capture the Mg isotopic effect observed in Grigoryan et al. [10,40,41]. It is also possible that if sampling in the present study was delayed beyond one hour post-meal, the effect observed in Grigoryan et al. may be reproduced as there is still active insulin action. Future work in this area should be complemented by the quantification of erythrocyte Mg^2+^ and determination of its Mg isotopic composition.

Ultimately, the consistent relative enrichment of ^24^Mg in the serum of individuals with T1DM across two studies provides evidence for the serum Mg isotopic composition as a trait of T1DM. Alternatively, the serum Mg isotopic composition may be a marker of Mg status, even in the absence of hypomagnesemia as defined by serum Mg concentration, but further work is required to identify the underlying isotopic fractionation mechanism.

### 3.2. Insulin Resistance in T1DM

It was previously suggested that the relative enrichment of ^24^Mg in the serum of individuals with T1DM may not be specific to the T1DM condition, but instead to phenotypes associated with insulin resistance preceding clinical disease conditions [25,28]. Insulin resistance is a hallmark of T2DM, and though less common in T1DM, can be observed during pubertal development and inter-current illness, and may also be related to obesity, sedentary lifestyle, family history of T2DM, and weight gain associated with intensive insulin therapy [42,43]. Whereas all T1DM participants in the present study are past the age of puberty and raised no confounding health issues during recruitment, eight out of 15 participants are overweight (body mass index (BMI) ≥ 25 kg m^−2^), and one is obese (BMI ≥ 30 kg m^−2^) [44,45]. However, the data do not suggest a relationship between BMI and δ^26^Mg, and most participants in this study likely required a daily insulin dose of ≤1.5 IU kg^−1^ (median pre-meal insulin dose in this study = 0.10 IU kg^−1^). Taking into account the above considerations, it seems unlikely that insulin resistance plays a role in Mg isotopic fractionation in T1DM [8].

### 3.3. Contributions of Mg from Muscle and Bone

The loss of skeletal muscle mass and decline in muscle function is a normal part of the aging process, but individuals with T1DM begin to exhibit diabetes-associated muscle decline at a much younger age [46]. Given the muscle decline and that the much larger exchangeable pool of Mg in muscle and other tissues is often drawn from to supplement blood Mg levels as required in order to maintain Mg homeostasis, it is possible that contributions from muscle Mg may influence serum Mg isotopic compositions in individuals with T1DM [15,23,24]. In support of this, muscle displayed the lowest δ^26^Mg value (−1.23 ± 0.10‰) of 11 fluids and organs measured in aged (40–65 weeks) male wild type mice and the second lowest δ^26^Mg value (−1.15 ± 0.05‰) in young (14–28 weeks) male wild-type mice [30,47]. It is not known whether the T1DM individuals from Grigoryan et al. experienced muscle mass loss, but this could be a ^24^Mg-enriched source contributing to low serum δ^26^Mg values in individuals with T1DM [25].

### 3.4. Correlation between Serum Mg Concentration and Isotopic Composition

**Figure 4:**
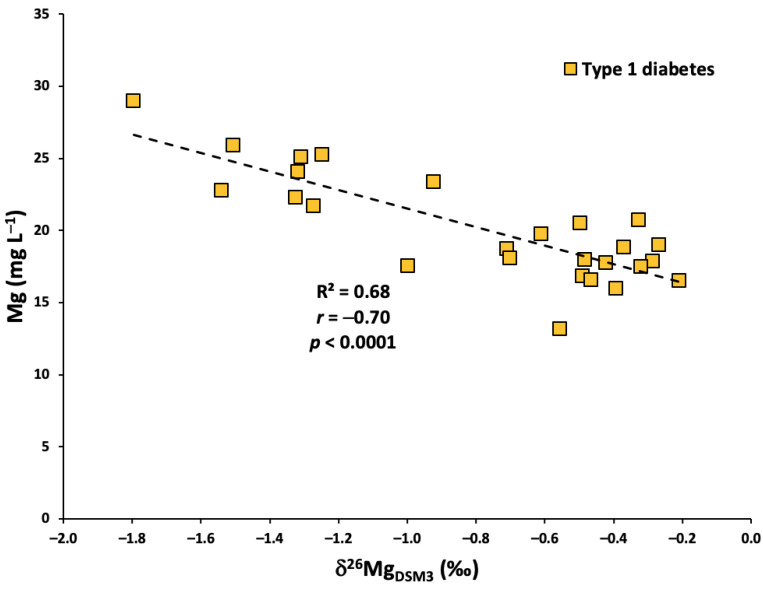
The relationship between serum Mg concentrations and δ^26^Mg in individuals with T1DM from Grigoryan et al. [25].

There is a lack of significant correlation between the serum Mg concentration and isotopic composition in all but one dataset between the present study and Grigoryan et al.—the exception being the T1DM group from Grigoryan et al., in which a highly significant inverse correlation was observed (*r* = −0.70, *p* < 0.0001) (Figure 4) [25]. The association of high serum Mg concentrations and low δ^26^Mg values also supports the theory of muscle as a Mg-rich, ^26^Mg-depleted Mg source in individuals with T1DM. However, the range of serum Mg concentrations determined in T1DM individuals from Grigoryan et al. (13.2 to 29.0 mg L^−1^) is almost identical to the range from this study (15.5 to 29.9 mg L^−1^), and this relationship was not observed in the present study, making the interpretation of this correlation a challenge.

### 3.5. Study Limitations

A limitation for the present study is that there are a number of factors that can influence Mg homeostasis, complicating the study of serum Mg dynamics [15]. Although all participants were provided the same meal, overall diet (trophic level of organism demonstrated to induce Mg, Ca, Cu, and Zn isotopic variations) was not controlled for and dietary habits were not recorded [39]. High phosphoric acid in soft drinks, a low protein diet (<30 mg day^−1^), and foods containing phytates, polyphenols, and oxalic acid (e.g., rice and nuts) have the ability to bind Mg and produce insoluble precipitates, reducing Mg bioavailability and absorption [48,49,50,51]. This is important because dietary differences have been shown to translate into changes in Mg isotopic compositions, with δ^26^Mg increasing from herbivores to higher-level consumers (i.e., secondary, tertiary, and above in the food chain) [52,53]. Therefore, omnivores would generally be expected to have a bodily Mg isotopic composition that is relatively enriched in ^26^Mg compared to vegetarians and vegans. Fluoride is commonly found in drinking water and other dietary staples, and prevents Mg absorption through binding and production of insoluble complexes [54,55,56]. Caffeine and alcohol increase renal excretion of Mg and common medications (e.g., antacids, antibiotics, oral contraceptives, and diuretics) have a negative effect on Mg absorption [15,57,58,59,60,61,62,63,64,65]. Further, the blood Mg concentration has been demonstrated to increase in response to Mg supplementation [23,24]. Other limitations include the relatively small sample sizes for the groups (control = 10; T1DM = 15) and the difference between their mean ages of 12 years. Finally, data were not collected on insulin resistance markers (TG levels, TG/HDL ratio, and total cholesterol/HDL ratio), which could have provided information on the link between insulin resistance and the disruption of Mg homeostasis in T1DM and test the hypothesis of Le Goff et al. [28,66].

## 4. Materials and Methods

### 4.1. Reagents and Materials

Blood samples were collected in Vacutainer BD SST™ ll Advance Tubes with Gel and Clot Activator (5 mL). Ultrapure water (resistivity > 18.2 MΩ cm) was obtained from a Milli-Q Element water purification system (Merck Millipore, Bedford, MA, USA). Trace metal analysis grade 14 M HNO_3_ and 12 M HCl (Fisher Chemicals, Leicestershire, UK) were further purified by sub-boiling distillation in a Savillex DST-4000 acid purification system (Savillex Corporation, Eden Prairie, MN, USA). Ultrapure TraceSELECT^®^ 9.8 M hydrogen peroxide and ACS grade acetone were purchased from Sigma Aldrich (Overijse, Belgium).

A multi-element solution containing Na, Mg, K, Ca, Fe, Cu and Zn was prepared from single-element standard solutions (1000 mg L^−1^) from Inorganic Ventures (Christiansburg, VA, USA) for quantification purposes. A 1000 mg L^−1^ Ga standard solution (Inorganic Ventures) was used as an internal standard for element quantification. A standard solution, CPI Mg (CPI International, lot 109778-46), was used as an in-house isotopic standard for monitoring instrument performance and the accuracy and precision of the Mg isotope ratios. The Seronorm™ Trace Elements Serum reference material (lot 1801802, SERO AS, Billingstad, Norway) was also used for method evaluation.

Two-mL polypropylene chromatographic columns (Eichrom Technologies, Saint Jacques de la Lande, France) and AG50W-X8 strong cation exchange resin (hydrogen form, 8% cross-linkage, 100–200 mesh size) purchased from Bio-Rad (Temse, Belgium) were used for Mg isolation.

### 4.2. Study Design

This study was reviewed and approved (BC-05376) by an independent Medical Ethics Committee associated with Ghent University Hospital and Ghent University, and all subjects signed an informed consent form concerning this study. The study was conducted according to the guidelines of good clinical practice (ICH/GCP) and the Declaration of Helsinki established to protect people participating in clinical studies.

Participants were recruited by the Ghent University Hospital. Ten apparently healthy male participants (control group) and 15 males with T1DM (identified based on clinical diagnosis) were recruited for this study. The average age of the control group was 35.1 ± 14.6 (1SD) years and the T1DM group was 47.7 ± 16.5 (1SD) years. Participants were asked to consume their usual breakfast at 8:00 and arrive at the Ghent University Hospital at 11:00 to provide their first blood sample. Age, weight, and height were recorded at this time (Tables S1 and S2). In the event of hypoglycemia ahead of sampling, participants consumed food as needed. Diabetic participants typically injected insulin immediately following first blood sampling and began their meal within ~30 min. A standard meal (556 kcal) consisting of 4 slices of bread, 2 slices of cheese, and one portion of butter was provided as a lunch. A second blood sample was drawn one hour after finishing the meal in order to integrate both the rise in glycemia due to gastro-intestinal absorption and the effects of insulin. Routine tests, such as for glycemic control (HbA1C) and the presence/absence of anemia, were performed on individuals with T1DM (Table S2). The blood samples were centrifuged to remove the clot and the serum (supernatant) samples were immediately stored at −20 °C until further sample preparation in a class-10 clean lab (PicoTrace™, Göttingen, Germany) at the Department of Chemistry of Ghent University.

### 4.3. Sample Preparation

Samples were thawed and aliquots of ~0.25 mL were digested in closed Savillex^®^ PFA beakers using 2 mL of 14 M HNO_3_ and 0.5 mL of 9.8 M H_2_O_2_ at 110 °C for 24 h. After cooling, the vessels were opened and the digests were evaporated to dryness at 90 °C. The residues were then dissolved in 1.2 mL of 0.4 M HCl for Mg isolation using cation exchange chromatography as described previously [25]. For determination of the Mg concentration, a small aliquot of each digested sample (in 0.4 M HCl) was taken, evaporated to dryness at 90 °C, and the residue dissolved in 0.3 M HNO_3_. Following isolation, samples were evaporated to dryness at 90 °C and dissolved in 0.3 M HNO_3_ for Mg isotopic analysis. Samples were further diluted for Mg quantification and isotope ratios measurement.

### 4.4. Instrumentation and Measurements

The Mg concentration was determined using an Agilent 8800 ICP-MS/MS instrument (ICP-QQQ, Agilent Technologies, Tokyo, Japan). To correct for signal drift, matrix effects, and instrument instability during element quantification, external calibration with Ga as internal standard (10 μg L^−1^) was used. The collision reaction cell was pressurized with the NH_3_/He gas mixture (9.93 ± 0.20 mol% in He, Mixture CRYSTAL, Air Liquide, Belgium) and pure He (99.9999%, ALPHAGAZ™ 2, Air Liquide, Belgium). The ^24^Mg nuclide was monitored in a mass shift approach (Q1 = 24, Q2 = 75, reaction product ion monitored: Mg(NH_3_)_3_^+^), whereas ^71^Ga was monitored on-mass [67]. Instrument operating conditions can be found in Table S3.

Magnesium isotope ratios (^26^Mg/^24^Mg and ^25^Mg/^24^Mg) were measured using a Thermo Scientific Neptune multi-collector inductively coupled plasma-mass spectrometry (MC-ICP-MS) instrument (Bremen, Germany) following the protocol reported by Grigoryan et al. [25]. Instrument operating conditions can be found in Table S4. The standard-sample bracketing (SSB) approach was used to correct for instrumental mass discrimination [68].

### 4.5. Statistical Analysis

Statistical analyses were performed using GraphPad Prism version 10.0.2 for Mac OS, GraphPad Software, Boston, MA, USA, www.graphpad.com (accessed on 8 October 2023). The normality of the datasets was tested using the Shapiro-Wilk’s test. To compare the control and T1DM groups, the Student’s *t*-test was used for parametric datasets, whereas the Mann-Whitney U test was used for non-parametric datasets. To evaluate any within-group differences before and after insulin injection and/or meal consumption, the paired *t*-test was used for parametric datasets, whereas the Wilcoxon matched-pairs signed-rank test was used for non-parametric datasets. The significance of relationships between variables was assessed using the Pearson correlation coefficient (parametric data) or the Spearman rank correlation coefficient (non-parametric data). If one of any two datasets being compared was non-parametric, the non-parametric test was employed. *p*-values of <0.05 were considered statistically significant.

## 5. Conclusions

This study Investigated the effect of administered insulin as a cause of temporal variability in the serum Mg isotopic composition through the comparison of blood serum samples collected before and one hour after insulin injection and meal consumption by T1DM individuals. The serum Mg isotopic response of healthy individuals after meal consumption was also examined. A significant difference in the serum Mg isotopic composition was found between T1DM individuals and controls, corroborating previous results [25]. This suggests that the disruption of Mg homeostasis persists in T1DM individuals despite only one out of 15 (6.7%) T1DM individuals in this study having a serum Mg concentration that is in the range of hypomagnesemia (<17.0 mg L^−1^). This is comparable rates of hypomagnesemia in the general population [8,11,12]. However, the average difference between pre- and post-insulin injection/meal T1DM samples of 0.05 ± 0.13‰ (1SD) was not significant (*p* = 0.18), indicating that the effect of administered insulin is not a cause of the temporal variability in the serum Mg isotopic composition previously observed in T1DM individuals [25]. No difference (*p* = 0.49) was observed for controls before (−0.12 ± 0.16‰) and after the meal (−0.10 ± 0.13‰), suggesting a lack of a postprandial Mg isotopic response within one hour of food consumption, and that the timing of the most recent meal may not require controlling for when determining serum Mg isotopic composition. Future work should investigate whether the large temporal Mg isotopic variations that were previously identified in the serum of T1DM individuals are captured by sampling during the period between insulin injection (when intracellular Mg^2+^ is increased) and meal consumption [25]. It is also possible that the large temporal Mg isotopic variations may be captured by delaying sampling beyond one hour post-meal as there is still active insulin action [25]. Future work in this area involving the analysis of Mg in serum should be complemented by the quantification of erythrocyte Mg^2+^ and determination of its Mg isotopic composition to elucidate the short-term Mg dynamics in blood following insulin injection.

## Figures and Tables

**Table 1:** Elemental and isotopic analysis of reference materials for method validation.

Material	n (m)	Mg ± 1SD (mg L^−1^)	n (m)	Measured δ^26^Mg_DSM3_ ± 2SD (‰)	Measured δ^25^Mg_DSM3_ ± 2SD (‰)	Reported δ^26^Mg_DSM3_ ± 2SD (‰)	Reported δ^25^Mg_DSM3_ ± 2SD (‰)	References
CPI Mg	-		25	−1.41 ± 0.11	−0.73 ± 0.06	−1.39 ± 0.08	−0.73 ± 0.04	[30]
Seronorm serum L-1	2(2)	20.1 ± 0.4	5(2)	0.08 ± 0.10	0.03 ± 0.05	0.12 ± 0.11	0.06 ± 0.06	[30]
						0.13 ± 0.11	0.06 ± 0.06	[31]
						0.08 ± 0.06	0.04 ± 0.05	[25]

n = number of measurements performed, m = number of separate aliquots digested. 1SD = standard deviation, 2SD = standard deviation multiplied by two.

**Table 2:** Magnesium concentration in blood serum obtained from participants before and one hour after the insulin injection (only for T1DM) and/or standard meal consumption.

Control Group				Type 1 Diabetes			
	Mg (mg L^−1^)		ΔMg (mg L^−1^)		Mg (mg L^−1^)		ΔMg (mg L^−1^)
Sample ID	Before	After	After—Before	Sample ID	Before	After	After—Before
MgG-1	25.2	25.1	−0.1	MgD-1	21.0	20.3	−0.7
MgG-2	21.9	24.7	2.8	MgD-2	18.5	17.6	−0.8
MgG-3	24.7	22.6	−2.1	MgD-3	17.1	21.1	4.0
MgG-4	28.5	23.1	−5.5	MgD-4	29.9	20.6	−9.3
MgG-5	20.7	20.7	0.0	MgD-5	21.9	21.1	−0.8
MgG-6	20.9	25.2	4.3	MgD-6	22.0	21.7	−0.3
MgG-7	23.2	23.0	−0.2	MgD-7	20.2	20.3	0.1
MgG-8	18.6	17.4	−1.3	MgD-8	21.2	21.8	0.6
MgG-9	20.4	19.6	−0.8	MgD-9	24.1	24.6	0.6
MgG-10	20.7	21.1	0.4	MgD-10	23.9	18.5	−5.3
				MgD-11	21.2	19.5	−1.7
				MgD-12	16.8	15.5	−1.3
				MgD-13	17.8	19.3	1.5
				MgD-14	18.8	18.7	−0.1
				MgD-15	18.6	17.7	−0.9
Mean	22.5	22.2	−0.2	Mean	20.9	19.9	−1.0
1SD	2.9	2.5	2.6	1SD	3.4	2.2	3.0
n	10	10		n	15	15	
1SE	0.9	0.8	0.8	1SE	0.9	0.6	0.8
Median	21.4	22.8	−0.2	Median	21.0	20.3	−0.7
Minimum	18.6	17.4	−5.5	Minimum	16.8	15.5	−9.3
Maximum	28.5	25.2	4.3	Maximum	29.9	24.6	4.0

ΔMg = Mg_after_ − Mg_before_. The typical precision on Mg concentration results as obtained with ICP-MS/MS was between 3 and 5%. The repeatability of the analytical procedure, expressed as the standard deviation of the Mg concentration determined for n = 2 aliquots of the same sample, did not exceed 1.3 mg L^−1^. 1SD = standard deviation, 1SE = standard error.

**Table 3:** δ^26^Mg value of blood serum drawn from participants one hour before and one hour after the insulin injection (only for T1DM) and/or standard meal consumption.

Control Group				Type 1 Diabetes			
	δ^26^Mg_DSM3_ (‰)		Δ^26^Mg (‰)		δ^26^Mg_DSM3_ (‰)		Δ^26^Mg (‰)
Sample ID	Before	After	After—Before	Sample ID	Before	After	After—Before
MgG-1	−0.21	−0.26	−0.05	MgD-1	−0.40	−0.39	0.00
MgG-2	−0.06	−0.03	0.03	MgD-2	−0.20	−0.10	0.10
MgG-3	−0.30	−0.25	0.05	MgD-3	−0.27	−0.23	0.04
MgG-4	−0.01	0.00	0.01	MgD-4	−0.30	0.00	0.30
MgG-5	−0.40	−0.28	0.12	MgD-5	−0.51	−0.24	0.27
MgG-6	−0.13	−0.17	−0.04	MgD-6	−0.45	−0.43	0.02
MgG-7	−0.16	−0.01	0.16	MgD-7	−0.36	−0.25	0.11
MgG-8	0.11	0.02	−0.09	MgD-8	0.00	−0.07	−0.07
MgG-9	0.08	0.03	−0.05	MgD-9	−0.15	−0.21	−0.06
MgG-10	−0.12	−0.01	0.11	MgD-10	−0.32	−0.38	−0.06
				MgD-11	−0.38	−0.33	0.06
				MgD-12	−0.27	−0.41	−0.14
				MgD-13	−0.11	0.02	0.13
				MgD-14	−0.40	−0.31	0.09
				MgD-15	−0.33	−0.43	−0.09
Mean	−0.12	−0.10	0.02	Mean	−0.30	−0.25	0.05
1SD	0.16	0.13	0.08	1SD	0.14	0.15	0.13
n	10	10		n	15	15	
1SE	0.05	0.04	0.03	1SE	0.04	0.04	0.03
Median	−0.12	−0.02	0.02	Median	−0.32	−0.25	0.04
Minimum	−0.40	−0.28	−0.09	Minimum	−0.51	−0.43	−0.14
Maximum	0.11	0.03	0.16	Maximum	0.00	0.02	0.30

Δ^26^Mg = δ^26^Mg_after_ − δ^26^Mg_before_. The internal precision, expressed as 2 times the standard error (2SE) for n = 45 measurements of the same sample, ranged from 0.01 to 0.02‰ (^26^Mg/^24^Mg). The repeatability, expressed as 2 times the standard deviation (2SD) for δ^26^Mg values determined in n = 2 aliquots of the same sample, ranged from 0.07 (sample MgD-1 after) to 0.10‰ (serum reference material). 1SD = standard deviation, 1SE = standard error.

## Data Availability

The data presented in this study are openly available in this article and the Supplementary Materials. Further information is available upon request from the corresponding author.

## Supplementary Materials

The following supporting information can be downloaded at: https://www.mdpi.com/article/10.3390/ijms242115683/s1.

## References

[1] WHO (2019). Classification of Diabetes Mellitus.

[2] WHO (2018). The Top 10 Causes of Death.

[3] Paschou S.A., Petsiou A., Chatzigianni K., Tsatsoulis A., Papadopoulos G.K. (2014). Type 1 Diabetes as an Autoimmune Disease: The Evidence. Diabetologia.

[4] Plowright A.T. (2015). Cardiovascular and Metabolic Disease: Scientific Discoveries and New Therapies. Edited by Philip Peplow, James Adams, Tim Young. ChemMedChem.

[5] Poretsky L. (2010). Principles of Diabetes Mellitus.

[6] Pickup J.C. (1994). Hypomagnesaemia in IDDM Patients with Microalbuminuria and Clinical Proteinuria. Diabetologia.

[7] McNair P., Christensen M.S., Christiansen C., Madsbad S., Transbøl I.B. (1982). Renal Hypomagnesaemia in Human Diabetes Mellitus: Its Relation to Glucose Homeostasis. Eur. J. Clin. Investig..

[8] Oost L.J., van Heck J.I., Tack C.J., de Baaij J.H. (2022). The Association between Hypomagnesemia and Poor Glycaemic Control in Type 1 Diabetes Is Limited to Insulin Resistant Individuals. Sci. Rep..

[9] Barbagallo M., Dominguez L.J. (2015). Magnesium and Type 2 Diabetes. World J. Diabetes.

[10] Delva P., Degan M., Trettene M., Lechi A. (2006). Insulin and Glucose Mediate Opposite Intracellular Ionized Magnesium Variations in Human Lymphocytes. J. Endocrinol..

[11] Van Dijk P.R., Waanders F., Qiu J., de Boer H.H., Van Goor H., Bilo H.J.G. (2020). Hypomagnesemia in Persons with Type 1 Diabetes: Associations with Clinical Parameters and Oxidative Stress. Ther. Adv. Endocrinol. Metab..

[12] Ahmed F., Mohammed A. (2019). Magnesium: The Forgotten Electrolyte—A Review on Hypomagnesemia. Med. Sci..

[13] Gerstl E.-M., Rabl W., Rosenbauer J., Gröbe H., Hofer S.E., Krause U., Holl R.W. (2008). Metabolic Control as Reflectet by HbA1c in Children, Adolescents and Young Adults with Type-1 Diabetes Mellitus: Combined Longitudinal Analysis Including 27,035 Patients from 207 Centers in Germany and Austria during the Last Decade. Eur. J. Pediatr..

[14] Rodrigues A.K., Melo A.E., Domingueti C.P. (2020). Association between Reduced Serum Levels of Magnesium and the Presence of Poor Glycemic Control and Complications in Type 1 Diabetes Mellitus: A Systematic Review and Meta-Analysis. Diabetes Metab. Syndr..

[15] Workinger J.L., Doyle R.P., Bortz J. (2018). Challenges in the Diagnosis of Magnesium Status. Nutrients.

[16] McCarthy J.T., Kumar R. (1999). Atlas of Diseases of the Kidney: Divalent Cation: Magnesium; Current Medicine.

[17] Hardwick L.L., Jones M.R., Brautbar N., Lee D.B. (1990). Site and Mechanism of Intestinal Magnesium Absorption. Miner. Electrolyte Metab..

[18] Elin R.J. (1987). Assessment of Magnesium Status. Clin. Chem..

[19] Gürlek A., Bayraktar M., Özaltin N. (1998). Intracellular Magnesium Depletion Relates to Increased Urinary Magnesium Loss in Type I Diabetes. Horm. Metab. Res..

[20] DiNicolantonio J.J., O’Keefe J.H., Wilson W. (2018). Subclinical Magnesium Deficiency: A Principal Driver of Cardiovascular Disease and a Public Health Crisis. Open Heart.

[21] Ising H., Bertschat F., Günther T., Jeremias E., Jeremias A. (1995). Measurement of Free Magnesium in Blood, Serum and Plasma with an Ion-Sensitive Electrode. Clin. Chem. Lab. Med..

[22] Blaine J., Chonchol M., Levi M. (2015). Renal Control of Calcium, Phosphate, and Magnesium Homeostasis. Clin. J. Am. Soc. Nephrol..

[23] Lim P., Jacob E., Dong S., Khoo O.T. (1969). Values for Tissue Magnesium as a Guide in Detecting Magnesium Deficiency. J. Clin. Pathol..

[24] Witkowski M., Hubert J., Mazur A. (2011). Methods of Assessment of Magnesium Status in Humans: A Systematic Review. Magnes. Res..

[25] Grigoryan R., Costas-Rodríguez M., Van Laecke S., Speeckaert M., Lapauw B., Vanhaecke F. (2019). Multi-Collector ICP-Mass Spectrometry Reveals Changes in the Serum Mg Isotopic Composition in Diabetes Type I Patients. J. Anal. At. Spectrom..

[26] Brand W.A., Coplen T.B., Vogl J., Rosner M., Prohaska T. (2014). Assessment of International Reference Materials for Isotope-Ratio Analysis (IUPAC Technical Report). Pure Appl. Chem..

[27] Hastuti A.A.M.B., Costas-Rodríguez M., Matsunaga A., Ichinose T., Hagiwara S., Shimura M., Vanhaecke F. (2020). Cu and Zn Isotope Ratio Variations in Plasma for Survival Prediction in Hematological Malignancy Cases. Sci. Rep..

[28] Le Goff S., Godin J.-P., Albalat E., Nieves J.M.R., Balter V. (2022). Magnesium Stable Isotope Composition, but Not Concentration, Responds to Obesity and Early Insulin-Resistant Conditions in Minipig. Sci. Rep..

[29] Le Goff S., Albalat E., Dosseto A., Godin J.-P., Balter V. (2021). Determination of Magnesium Isotopic Ratios of Biological Reference Materials via Multi-Collector Inductively Coupled Plasma Mass Spectrometry. Rapid Commun. Mass Spectrom..

[30] Grigoryan R., Costas-Rodríguez M., Vandenbroucke R.E., Vanhaecke F. (2020). High-Precision Isotopic Analysis of Mg and Ca in Biological Samples Using Multi-Collector ICP-Mass Spectrometry after Their Sequential Chromatographic Isolation—Application to the Characterization of the Body Distribution of Mg and Ca Isotopes in Mice. Anal. Chim. Acta.

[31] De Vega C.G., Chernonozhkin S.M., Grigoryan R., Costas-Rodríguez M., Vanhaecke F. (2020). Characterization of the new isotopic reference materials IRMM-524A and ERM-AE143 for Fe and Mg isotopic analysis of geological and biological samples. J. Anal. At. Spectrom..

[32] Vogl J., Brandt B., Noordmann J., Rienitz O., Malinovskiy D. (2016). Characterization of a Series of Absolute Isotope Reference Materials for Magnesium: Ab Initio Calibration of the Mass Spectrometers, and Determination of Isotopic Compositions and Relative Atomic Weights. J. Anal. At. Spectrom..

[33] Wallace T.M., Matthews D.R. (2002). The Assessment of Insulin Resistance in Man. Diabet. Med..

[34] Pickup J., Williams G. (1997). Textbook of Diabetes.

[35] Gragossian A., Bashir K., Bhutta B.S., Friede R. (2023). Hypomagnesemia.

[36] Odusan O.O., Familoni O.B., Odewabi A.O., Idowu A.O., Adekolade A.S. (2017). Patterns and Correlates of Serum Magnesium Levels in Subsets of Type 2 Diabetes Mellitus Patients in Nigeria. Indian J. Endocrinol. Metab..

[37] Sullivan K., Moore R.E.T., Rehkämper M., Layton-Matthews D., Leybourne M.I., Puxty J., Kyser T.K. (2020). Postprandial Zinc Stable Isotope Response in Human Blood Serum. Metallomics.

[38] Lauwens S., Costas-Rodríguez M., Van Vlierberghe H., Vanhaecke F. (2017). High-Precision Isotopic Analysis of Cu in Blood Serum via Multi-Collector ICP-Mass Spectrometry for Clinical Investigation: Steps towards Improved Robustness and Higher Sample Throughput. J. Anal. At. Spectrom..

[39] Sullivan K.V., Moore R.E.T., Vanhaecke F. (2023). The Influence of Physiological and Lifestyle Factors on Essential Mineral Element Isotopic Compositions in the Human Body: Implications for the Design of Isotope Metallomics Research. Metallomics.

[40] Kolisek M., Sponder G., Pilchova I., Cibulka M., Tatarkova Z., Werner T., Racay P., de Tombe P., Gudermann T., Jahn R., Lill R. (2019). Magnesium Extravaganza: A Critical Compendium of Current Research into Cellular Mg2+ Transporters Other than TRPM6/7. Reviews of Physiology, Biochemistry and Pharmacology 176.

[41] Mastrototaro L., Tietjen U., Sponder G., Vormann J., Aschenbach J.R., Kolisek M. (2015). Insulin Modulates the Na^+^/Mg^2+^ Exchanger SLC41A1 and Influences Mg^2+^ Efflux from Intracellular Stores in Transgenic HEK293 Cells. J. Nutr..

[42] Priya G., Kalra S. (2018). A Review of Insulin Resistance in Type 1 Diabetes: Is There a Place for Adjunctive Metformin?. Diabetes Ther..

[43] Kaul K., Apostolopoulou M., Roden M. (2015). Insulin Resistance in Type 1 Diabetes Mellitus. Metabolism.

[44] Kruschitz R., Wallner-Liebmann S.J., Hamlin M.J., Moser M., Ludvik B., Schnedl W.J., Tafeit E. (2013). Detecting Body Fat–A Weighty Problem BMI versus Subcutaneous Fat Patterns in Athletes and Non-Athletes. PLoS ONE.

[45] Gallagher D., Heymsfield S.B., Heo M., Jebb S.A., Murgatroyd P.R., Sakamoto Y. (2000). Healthy Percentage Body Fat Ranges: An Approach for Developing Guidelines Based on Body Mass Index. Am. J. Clin. Nutr..

[46] Travis C., Srivastava P.S., Hawke T.J., Kalaitzoglou E. (2022). Diabetic Bone Disease and Diabetic Myopathy: Manifestations of the Impaired Muscle-Bone Unit in Type 1 Diabetes. J. Diabetes Res..

[47] Grigoryan R., Costas-Rodríguez M., Van Wonterghem E., Vandenbroucke R.E., Vanhaecke F. (2021). Effect of Endotoxemia Induced by Intraperitoneal Injection of Lipopolysaccharide on the Mg Isotopic Composition of Biofluids and Tissues in Mice. Front. Med..

[48] Bohn T. (2008). Dietary Factors Influencing Magnesium Absorption in Humans. Curr. Nutr. Food Sci..

[49] Philipp Schuchardt J., Hahn A. (2017). Intestinal Absorption and Factors Influencing Bioavailability of Magnesium-an Update. Curr. Nutr. Food Sci..

[50] Schwartz R., Walker G., Linz M.D., MacKellar I. (1973). Metabolic Responses of Adolescent Boys to Two Levels of Dietary Magnesium and Protein. I. Magnesium and Nitrogen Retention. Am. J. Clin. Nutr..

[51] Bohn T., Davidsson L., Walczyk T., Hurrell R.F. (2004). Phytic Acid Added to White-Wheat Bread Inhibits Fractional Apparent Magnesium Absorption in Humans. Am. J. Clin. Nutr..

[52] Martin J.E., Vance D., Balter V. (2015). Magnesium Stable Isotope Ecology Using Mammal Tooth Enamel. Proc. Natl. Acad. Sci. USA.

[53] Martin J.E., Vance D., Balter V. (2014). Natural Variation of Magnesium Isotopes in Mammal Bones and Teeth from Two South African Trophic Chains. Geochim. Cosmochim. Acta..

[54] Barker L.K., Duchon K.K., Lesaja S., Robison V.A., Presson S.M. (2017). Adjusted Fluoride Concentrations and Control Ranges in 34 States: 2006–2010 and 2015. J. Am. Water Works Assoc..

[55] Machoy-Mokrzynska A. (1995). Fluoride-Magnesium Interaction. Fluoride.

[56] Ersoy I.H., Koroglu B.K., Varol S., Ersoy S., Varol E., Aylak F., Tamer M.N. (2011). Serum Copper, Zinc, and Magnesium Levels in Patients with Chronic Fluorosis. Biol. Trace Elem. Res..

[57] Rylander R., Megevand Y., Lasserre B., Amstutz W., Granbom S. (2001). Moderate Alcohol Consumption and Urinary Excretion of Magnesium and Calcium. Scand. J. Clin. Lab..

[58] Kynast-Gales S.A., Massey L.K. (1994). Effect of Caffeine on Circadian Excretion of Urinary Calcium and Magnesium. J. Am. Coll. Nutr..

[59] William J.H., Danziger J. (2016). Magnesium Deficiency and Proton-Pump Inhibitor Use: A Clinical Review. J. Clin. Pharmacol..

[60] Begley J., Smith T., Barnett K., Strike P., Azim A., Spake C., Richardson T. (2016). Proton Pump Inhibitor Associated Hypomagnasaemia-a Cause for Concern?. Br. J. Clin. Pharmacol..

[61] Polk R.E. (1989). Drug-Drug Interactions with Ciprofloxacin and Other Fluoroquinolones. Am. J. Med..

[62] Dante G., Vaiarelli A., Facchinetti F. (2014). Vitamin and Mineral Needs during the Oral Contraceptive Therapy: A Systematic Review. Int. J. Reprod. Contracept. Obstet. Gynecol..

[63] Akinloye O., Adebayo T.O., Oguntibeju O.O., Oparinde D.P., Ogunyemi E.O. (2011). Effects of Contraceptives on Serum Trace Elements, Calcium and Phosphorus Levels. West Indian Med. J..

[64] Dørup I. (1994). Magnesium and Potassium Deficiency. Its Diagnosis, Occurrence and Treatment in Diuretic Therapy and Its Consequences for Growth, Protein Synthesis and Growth Factors. Acta Physiol. Scand. Suppl..

[65] Lim P., Jacob E. (1972). Magnesium Deficiency in Patients on Long-Term Diuretic Therapy for Heart Failure. Br. Med. J..

[66] Bîcu M.L., Bîcu D., Vladu M.I., Clenciu D., Chirila A.M.C., Vîlvoi D., Sandu M., Panduru N.M., Moța E., Moța M. (2015). Insulin Resistance Markers in Type 1 Diabetes Mellitus. Rom. J. Diabetes Nutr. Metab. Dis..

[67] Bolea-Fernandez E., Rua-Ibarz A., Resano M., Vanhaecke F. (2021). To Shift, or Not to Shift: Adequate Selection of an Internal Standard in Mass-Shift Approaches Using Tandem ICP-Mass Spectrometry (ICP-MS/MS). J. Anal. At. Spectrom..

[68] Yang L., Tong S., Zhou L., Hu Z., Mester Z., Meija J. (2018). A Critical Review on Isotopic Fractionation Correction Methods for Accurate Isotope Amount Ratio Measurements by MC-ICP-MS. J. Anal. At. Spectrom..

