# Supplementary material for: Serum Mg Isotopic Composition Reveals That Mg Dyshomeostasis Remains in Type 1 Diabetes despite the Resolution of Hypomagnesemia
Source: Int J Mol Sci. 2023 Oct 27;24(21):15683. doi: 10.3390/ijms242115683 (PMC10647222; doi:10.3390/ijms242115683)
Supplement: Supplementary file 1 [file ijms-24-15683-s001.zip › ijms-2624394-supplementary.docx]

Supplementary materials to:

**Serum Mg Isotopic Composition Reveals That Mg Dyshomeostasis Remains in Type 1 Diabetes despite the Resolution of Hypomagnesemia**

Kaj Vaughan Sullivan ^1^, Yasmina Assantuh ^1^, Rosa Grigoryan ^2^, Marta Costas-Rodríguez ^1,3^,
Eduardo Bolea-Fernandez ^1,4^, Bruno Lapauw ^5,6^, Steven Van Laecke ^7^ and Frank Vanhaecke ^1,^*

^1^ Atomic and Mass Spectrometry—A&MS Research Unit, Department of Chemistry, Ghent University, 9000 Ghent, Belgium; kaj.sullivan@ugent.be (K.V.S.); yasmina.assantuhazzouz@ugent.be (Y.A.); martacr@uvigo.gal (M.C.-R.); ebolea@unizar.es (E.B.-F.)

^2^ The Isotoparium, Division of Geological and Planetary Sciences, California Institute of Technology, Pasadena, CA 91125, USA; grigoryanrosa@gmail.com

^3^ Centro de Investigación Mariña, Universidade de Vigo, Departamento de Química Analítica y Alimentaria, Grupo QA2, 36310 Vigo, Spain

^4^ Department of Analytical Chemistry, Aragón Institute of Engineering Research (I3A), University of Zaragoza, 50009 Zaragoza, Spain

^5^ Department of Endocrinology, Ghent University Hospital, 9000 Ghent, Belgium; bruno.lapauw@uzgent.be

^6^ Department of Internal Medicine and Pediatrics, Ghent University, 9000 Ghent, Belgium

^7^ Renal Division, Department of Internal Medicine and Pediatrics, Ghent University Hospital, 9000 Ghent, Belgium; steven.vanlaecke@ugent.be

***** Correspondence: frank.vanhaecke@ugent.be

| **Table S1.** Control group physiological data. | | | |  |
| --- | --- | --- | --- | --- |
| **Sample ID** | **Age** | **Height (cm)** | **Weight (kg)** | **BMI (kg m^-2^)** |
| MgG-1 | 56 | 170 | 74 | 25.6 |
| MgG-2 | 55 | 179 | 88 | 27.5 |
| MgG-3 | 19 | 183 | 67 | 20.0 |
| MgG-4 | 26 | 177 | 95 | 30.3 |
| MgG-5 | 25 | 185 | 101 | 29.5 |
| MgG-6 | 30 | 175 | 67 | 22.0 |
| MgG-7 | 32 | 181 | 83 | 25.4 |
| MgG-8 | 56 | 175 | 76 | 24.8 |
| MgG-9 | 25 | 199 | 83 | 21.0 |
| MgG-10 | 27 | 182 | 76 | 22.9 |

| **Table S2.** Type 1 diabetes physiological data and clinical parameters. | | | | | | | | | | | | | |
| --- | --- | --- | --- | --- | --- | --- | --- | --- | --- | --- | --- | --- | --- |
| **Sample ID** | **Age** | **Height (cm)** | **Weight (kg)** | **BMI**  **(kg m^-2^)** | **Glycemia before (mg dL^-1^)** | **Glycemia after (mg dL^-1^)** | **Insulin** | **Insulin analog** | **Units** | **Serum creatinine (mg dL^-1^)** | **Hemoglobin (g dL^-1^)** | **HbA1c (%)** | **CRP**  **(mg L^-1^)** |
| **MgD-1** | 68 | 165 | 62 | 22.7 | 196 | 191 | Fiasp | Aspart | 4 IU | 0.82 | 14.5 | 6.2 | <1.0 |
| **MgD-2** | 62 | 174 | 80 | 26.4 | 165 | 185 | Humalog | Lispro | 4 IU | 0.93 | 14.8 | 8.2 | 1.7 |
| **MgD-3** | 25 | 177 | 93 | 29.5 | 202 | 231 | Novorapid | Aspart | 16 IU | - | - | - | - |
| **MgD-4** | 49 | 180 | 81 | 25.0 | 87 | 193 | Novorapid | Aspart | 7 IU | 0.79 | 13.5 | 9.3 | 3.6 |
| **MgD-5** | 54 | 170 | 74 | 25.6 | 69 | 209 | Novorapid | Aspart | 4 IU | 1.12 | 11.6 | 6.4 | <1.0 |
| **MgD-6** | 63 | 183 | 96 | 28.7 | 76 | 104 | Humalog | Lispro | 7 IU | 1.20 | 15.4 | 7.5 | 1.1 |
| **MgD-7** | 48 | 182 | 79 | 23.8 | 125 | 132 | Fiasp | Aspart | 8 IU | 0.89 | 14.8 | 7.8 | 3.5 |
| **MgD-8** | 58 | 178 | 84 | 26.5 | 107 | 224 | Novorapid | Aspart | 7 IU | 0.53 | 14.3 | 6.1 | 1.1 |
| **MgD-9** | 62 | 185 | 103 | 29.9 | 74 | 125 | Novorapid | Aspart | 14 IU | 1.09 | 15.2 | 7.3 | <1.0 |
| **MgD-10** | 24 | 167 | 60 | 21.3 | 207 | 221 | Fiasp | Aspart | 11 IU | 1.05 | 15.5 | 9.6 | 7.0 |
| **MgD-11** | 22 | 180 | 79 | 24.4 | 55 | 174 | Novorapid | Aspart | 3 IU | 1.05 | 16.5 | 6.5 | 2.6 |
| **MgD-12** | 70 | 172 | 73 | 24.7 | 192* | 309 | Fiasp | Aspart | 7 IU | 0.94 | 13.6 | 7.6 | 1.6 |
| **MgD-13** | 34 | 172 | 83 | 28.1 | 246 | 210 | Novorapid | Aspart | 35 IU | 0.94 | 17.2 | 7.5 | 2.1 |
| **MgD-14** | 35 | 180 | 81 | 24.8 | 63 | 167 | Humalog | Lispro | 8 IU | 1.06 | 14.8 | 8.2 | <1.0 |
| **MgD-15** | 41 | 183 | 123 | 36.7 | 174 | 172 | Lyumjev | Lispro | 15 IU | 0.86 | 14.1 | 7.8 | 4.7 |
| HbA1c = hemoglobin A1c. *Patient consumed banana two hours before providing blood sample due to hypoglycemia. - = data not available. | | | | | | | | | | | | | |

| **Table S3.** Agilent 8800 ICP-MS/MS operating conditions. | |
| --- | --- |
| Instrument settings | |
| Sample uptake rate | 0.35 mL min^-1^ |
| Plasma gas flow rate | 15.0 L Ar min^-1^ |
| Auxiliary gas flow rate | 0.9 L Ar min^-1^ |
| Nebulizer gas flow rate | ~1.12 L Ar min^-1^ |
| Collision gas flow rate | 1.0 mL He min^-1^ |
| Reaction gas flow rate | 3.0 mL NH_3_/He (10%/90%) min^-1^ |
| RF power | 1550 W |
| Sample cone | Ni tip with Cu base |
| Skimmer cone | Ni |
| Data acquisition parameters | |
| Integration time | 1 s |
| Replicates | 10 |
| Sweeps | 100 |
| Nuclides monitored | ^24^Mg^+^ → ^24^Mg(^14^N^1^H_3_)_3_^+^, ^71^Ga^+^ (on mass) |

| **Table S4.** Neptune MC-ICP-MS operating conditions. | |
| --- | --- |
| Instrument settings | |
| Forward power | 1200 W |
| Plasma gas flow rate | 15 L Ar min^-1^ |
| Auxiliary gas flow rate | 0.70 to 0.95 L Ar min^-1^ |
| Nebulizer gas flow rate | ~1 L Ar min^-1^ |
| Sample cone | Nickel, Jet-type: 1.1 mm orifice diameter |
| Skimmer cone | Nickel, X-type: 0.8 mm orifice diameter |
| Sample uptake | 0.1 mL min^-1^ |
| Mass resolution mode | Low |
| Data acquisition parameters | |
| Faraday cup configuration/amplifier | L3 (^24^Mg)/10^11^ Ω, C (^25^Mg)/10^11^ Ω, H3 (^26^Mg)/10^11^ Ω |
| Sensitivity | 16 V for ^24^Mg at 150 ng g^-1^ |
| Signal integration time | 2.097 s |
| No. of integrations, blocks, cycles/block | 3, 1, 45 |
| Blank signal (2% HNO_3_) | 0.01V for ^24^Mg |
